# Peri-implant bone-level changes in the second decade of loading with regard to the implant–abutment connection: a retrospective study on implants under systematic aftercare

**DOI:** 10.1186/s40729-021-00384-1

**Published:** 2021-09-27

**Authors:** Anne Brigitte Kruse, Vanessa Wild, Petra Ratka-Krüger, Kirstin Vach, Eberhard Frisch

**Affiliations:** 1 Department of Operative Dentistry and Periodontology, Faculty of Medicine, University of Freiburg, Hugstetter Str. 55, 79106 Freiburg, Germany; 2 Zahn Service Center Stuttgart, Charlottenplatz 6, 70173 Stuttgart, Germany; 3 Institute of Medical Biometry and Statistics, Faculty of Medicine , University of Freiburg, Zinkmattenstr. 6A, 79108 Freiburg, Germany; 4 Northern Hessia Implant Center, Industriestr. 17A, 34369 Hofgeismar, Germany

**Keywords:** Dental implant, Bone loss, Bone-level changes, Implant–abutment connection, Supportive implant therapy, Long-term results

## Abstract

**Background:**

This retrospective study investigates the change in the peri-implant bone level (PBL) during the 2nd decade of intraoral function in patients complying with a ‘supportive implant therapy’ (SIT) program. The results were statistically analyzed with respect to the implant abutment connection used.

**Methods:**

In a private practice, only patients with 20-year SIT compliance were identified. Of these, all patients with 10- and 20-year radiographs available were selected. Therefore, no control group was possible and implant losses had to be excluded. Two experienced researchers assessed the peri-implant bone levels. As three different abutment connection concepts (bone-level butt-joint, bone-level conical and tissue-level conical) and two different implant surfaces (machined vs. roughened) were involved, statistical analyses were performed to detect potential differences.

**Results:**

Ninety-three implants from 36 patients with 20-year SIT compliance and available radiographs were included in the study. At study baseline (10 years intraoral), a mean bone loss of − 1.7 mm (median − 1.2; standard deviation [sd] 1.4, range: 0 to − 7.2) was recorded. After 20 years, we found a mean bone loss of − 2.5 mm (median − 2.3, sd 1.79, range: − 0.5 to + 7.4). Furthermore, we found a mean bone loss of 0.8 mm in intraoral function from year 10 to year 20 (mean: 0.08 mm per year); this change was independent of the abutment connection type.

**Conclusions:**

During the 2nd decade of function, peri-implant bone loss in patients with SIT compliance might be small in value and should not be expected in all implants.

## Background

In recent decades, dental implants have become an indispensable treatment option for dental rehabilitation after tooth loss. Today, irrespective of implant design, surface treatment, diameters, implant**–**abutment connection design and bone quality, dental implants offer highly predictable solutions for replacing missing teeth [1–3]. Nonetheless, after prosthesis fixation, each implant is exposed to the microbial biofilm and the person might develop peri-implant tissue inflammation and, consequently, develop peri-implant bone loss. This disease known as named peri-implantitis has been defined as a pathological condition occurring in tissues around dental implants, characterized by inflammation in the peri‐implant connective tissue and a progressive loss of supporting bone [4]. Peri-implantitis occurs due to a disruption of the host–microbe homeostasis between the microbial challenge and the human host response at the implant**–**mucosa interface [5]. Some authors favorize a different explanatory model for peri-implantitis. They consider the implant as a foreign body that initiates a foreign body inflammatory reaction resulting in either total rejection or bony or fibrous tissue encapsulation [6].

The onset of peri‐implantitis may occur early during follow-up, and the disease typically progresses in a nonlinear and accelerating pattern [4, 7]. If untreated, peri-implantitis may finally lead to implant loss. A recent systematic review and meta-analysis comprising 47 studies revealed a weighted mean implant-based peri-implantitis prevalence of 9.25% [8]. Another study investigated randomly chosen Swedish patients with dental implants. Within a mean functional period of 9 years, the incidence for peri-implantitis was 45% [9]. One of the first 25-year follow-up studies of dental implants revealed a prevalence and incidence for peri-implantitis of 7% and 41%, respectively [10]. The findings of several studies emphasize the relevance of systematic postimplant aftercare programs and supportive implant therapies (SIT) designed to facilitate the prevention and early diagnosis of peri-implantitis [11–14]. As peri-implant bone-level changes have been identified to be a crucial parameter in the diagnosis of peri-implantitis, repeated radiographs in SIT seem to be essential. Consequently, the amount of peri-implant bone loss over time that could be considered normal should be defined. Unfortunately, the available data in the scientific literature are very limited to date, especially concerning long-term follow-up. Albrektsson et al. considered initial bone loss of < 1.5 mm in the first year after implant placement to be satisfactory [15]. After this distinction, an annual peri-implant bone loss of < 0.2 mm was considered not pathologic. In the following years, other researchers published different bone loss values: 2 mm within the first year [16], 2.5 mm after 5 years [17], and 1**–**2 mm as a threshold for the diagnosis of peri-implantitis [18]. In a recent study, Derks et al. followed patients with a total of 105 implants diagnosed with ‘moderate/severe peri-implantitis’ for a mean of 8.6 years and found mean peri-implant bone loss values of 0.4 mm/year [7]. To date, there has been an ongoing discussion on the amount of peri-implant bone loss that has to be considered as physiologically related to the different types of implant**–**abutment connection concepts (conical vs. butt-joint and tissue level vs. bone level) and also regarding the surface characteristics of the implant shoulder and perhaps the implant body.

Addressing the extensive lack of data on long-term peri-implant tissue-level alteration, the objective of this study is to evaluate the peri-implant bone-level changes during the 2nd decade of intraoral function and to evaluate whether significant differences can be detected among three different implant–abutment connection types (BL conical [BLC; morse-taper, Ankylos©], BL butt-joint [BLB; external hex, Branemark©]), and tissue-level conical [TLC; internal, ITI Bonefit©]).

## Methods

This retrospective study was conducted in a private practice specializing in dental implant therapy. A retrospective noninterventional study design was used based on the analysis of primary patient data that had been extracted from the patients’ records. We evaluated the radiological data of implants after 10 years and after 20 years. This study has been independently reviewed and approved by the Ethics Commission of Landeszahnärztekammer Hessen (No: 03/2020). The study was conducted in compliance with the appropriate EQUATOR guidelines (STROBE).

### Study population

The present study assessed data exclusively from implants under systematic postimplant aftercare (SIT) over 20 years. Patients who had received dental implants and implant-supported prostheses in our center and who had been compliant with our SIT program for > 20 years were identified (*n* = 60, with 184 implants; 35 females/25 males; mean age at final examination: 70.7 years). The SIT program includes regular visits (at least 1 appointment per year) to monitor oral hygiene indices and assessments of the peri-implant status in terms of bleeding and probing depths. After patients are instructed on how to perform and encouraged to perform at-home plaque control, a professional cleaning of the implant is performed. For further information on SIT see the study published by Frisch et al. 2020 [10]. These patients were approached and were asked to participate in the study after receiving written information regarding the aims and course of the investigation. Patients who provided written informed consent and met the inclusion criteria below were included.

- Age ≥ 18 years;
- Dental implants and implant-supported prostheses received at the study center;
- Observational period > 20 years;
- Availability of the complete medical history, including the following potential risk factors: medication, diabetes, cardiovascular disease (updated in 2-year intervals), in addition to smoking habits and a known history of periodontitis (in relevant cases, periodontitis was either treated or patients were edentulous);
- Availability of radiographs after both 10 years and after 20 years;
- Implant types: BLC, BLB, or TLC.

The exclusion criteria were as follows:

- No availability of analyzable radiographs after 10 years or after 20 years;
- Implant failure.

### Data collection

Between April 1 and October 1, 2020, the records of the patients in our study were evaluated according to the following parameters using patient records: age and sex, medical history, smoking habits, anatomical position of the implants (according to the Federation Dentaire Internationale [FDI] scheme), history of periodontitis, loss of implants and the period of observation. Moreover, during the 20-year appointment, the subjects were clinically examined by an experienced researcher (HF) who conducted a peri-implant examination (including probing depth (PD) and bleeding on probing (BOP)) for all implants. To assess the peri-implant bone level, intraoral radiographs were taken at 5-year intervals according to our SIT radiograph scheme. For the present study, we compared the 10- and 20-year radiographs obtained using an X-ray film holder in the parallel technique (Device: Heliodent DS; Sirona Dental Systems, Bensheim Germany).

### Data analysis

All radiographs were digitized and analyzed using a PC program (Sidexis XG, Sirona Dental Systems GmbH, Bensheim, Germany). To account for the anatomic magnification and distortion in the films, the linear dimensions of the images were calibrated. Based on the original implant length, this was achieved by setting the scale in the image to the known distance between the implant shoulder at the most apical point of the implant. An independent oral surgeon with a high level of expertise in image analysis who was not involved in other aspects of the study performed the initial radiographic examination under fourfold digital magnification. All measurements were saved and independently confirmed by another experienced periodontist other than the authors. The authors and the other periodontist assessed the radiographs, and in cases that differed in assessment outcomes, they reached a consensus value that was recorded. The 10-year bone loss values were calculated compared to the radiographs taken after implant insertion.

The present study included three different implant systems with technically different implant–abutment concepts: BL conical (BLC; morse-taper, Ankylos©), BL butt-joint (BLB; external hex, Branemark©), and tissue-level conical (TLC; internal, ITI Bonefit©). The BLB implants had a machined even surface, whereas the other implants (BLC and TLC) had roughened surfaces.

### Diagnostic criteria

During the present study, the following criteria for a diagnosis of peri-implantitis were used according to the ‘Consensus report of workgroup 4 of the 2017 World Workshop on the Classification of Periodontal and Peri‐Implant Diseases and Conditions’ [19]:

- Clinical signs of inflammation of the peri-implant soft tissues (redness, swelling, BOP+, and suppuration);
- Increased PD compared to the PD observed in previous examinations; and
- Radiographically, progredient bone loss beyond crestal peri-implant bone-level changes resulting from initial bone remodeling, and also possible bone gain.
- Implant survival was defined as an ‘osseointegrated implant in the oral cavity irrespective of the peri-implant tissue conditions’.
- Implant success was defined as ‘no signs of peri-implantitis during the entire observational period’.

### Treatment of peri-implantitis

In cases of a positive diagnosis of peri-implantitis, these patients were recommended for our three-step treatment plan. The 3 steps were as follows: (1) A nonsurgical approach (repeated biofilm removal and instillation of chlorhexidine gluconate (CHX)) over a 3-month period; (2) Examination of the peri-implant soft tissue architecture (tissue thickness, keratinized mucosa (KM) width, tissue mobility) and, if necessary, KM augmentation surgery via a free gingival graft (FGG) or a partially epithelialized free connective tissue graft (PECTG) [20]; and (3) Regenerative peri-implant bone surgery (ß-Tricalcium-phosphate plus resorbable membranes).

### Statistical analysis

Means, medians and standard deviations were calculated for descriptive analysis of the data. Linear mixed models with random intercepts were fitted for each patient to assess group (implant type, abutment designs, implant surfaces, peri-implantitis) effects on the response variables (mean 10-year PBL, 20-year PBL; and change between 10 and 20 year). For additional pairwise comparisons, the Bonferroni method was applied to correct for the multiple testing problem (adjustment of *p* values).

The calculations were performed with the statistical software STATA 16.1 (StataCorp LT, College Station, TX, USA) using “xtmixed”. The probability level for statistical significance was set to *p* < 0.05.

## Results

We identified 60 patients (184 implants) with SIT compliance over an observational period of > 20 years. Of these, 49 implants (no radiographs after 10 years) and 42 implants (no radiographs after 20 years) had to be excluded. Thirteen implants were lost during the 20-year period. Therefore, 36 patients (21 females and 15 males) with 93 implants and a mean age of 70 years (median 72 years, standard deviation [sd] 11 years) were included in the study. The sample included 4 smokers, 2 patients with diabetes, 22 individuals who suffered from cardiovascular diseases and 26 individuals who had a known history of periodontitis. The pertinent patient data are displayed in Table 1.

**Table 1:** Characteristics of the investigated patients

			Total [*n* = 36]
Age in years (mean ± sd; median)			69.7 ± 10.6; 71.9
Sex [*n*]		Female	21 (58%)
		Male	15 (42%)
General illnesses		Diabetes mellitus	2 (6%)
		Coronary heart disease	22 (61%)
Tobacco smoker			4 (11%)
Known history of periodontitis			26 (72%)
Implants [*n* = 93]	Jaw	Maxilla [*n*, (%)]	48 (52%)
		Mandible [*n*, (%)]	45 (48%)

The anatomical distribution of the implants is shown in Table 2, and the distribution of the implant systems used is shown in Table 3.

**Table 2:** Distribution of the implant systems used

Implant system	Implant/abutment connection	*n*
Ankylos©	Bone-level conical (morse-taper)	38 (41%)
Branemark©	Bone-level butt-joint (external hex)	41 (44%)
ITI Bonefit©	Tissue-level conical (internal)	14 (15%)

**Table 3:** Anatomical distribution of included implants [*n* = 93] according to the FDI scheme

Number of implants maxilla [*n* = 48]	0	2	3	4	4	3	4	1	2	3	5	7	4	2	3	1
Tooth position (FDI)	18	17	16	15	14	13	12	11	21	22	23	24	25	26	27	28
	48	47	46	45	44	43	42	41	31	32	33	34	35	36	37	38
Number of implants mandibula [*n* = 45]	0	7	5	2	3	3	0	1	1	1	3	4	5	4	6	0

The frequencies of different prosthetic treatments (single crown, bridge or removable prosthesis) are shown in Table 4.

**Table 4:** Type of prosthesis on included implants [*n* = 93]

Prosthetic treatment	*n*
Single crown	20 (21.5%)
Bridge	52 (56%)
Removable prosthesis	21 (22.5%)

### First investigation (10 years)

The mean bone loss at study baseline (10 years of intraoral function) was 1.7 mm (median 1.2, sd 1.4) (Table 5).

**Table 5:** Peri-implant bone loss after 10 years

Implant type	*n*	Min. bone loss	Max. bone loss	Median	Mean	sd
Tissue-level conical	14	0.2	2.4	1.0	1.2	0.8
Bone-level butt-joint	41	0	7.2	1.0	1.7	1.6
Bone-level conical	38	0	5.3	1.7	1.9	1.4
Total	93	0	7.2	1.2	1.7	1.4

Presentation of minimal/maximal bone loss values at baseline (10 years) according to implant type

*sd*  standard deviation

### Final investigation (20 years)

The mean bone loss after 20 years of intraoral function was 2.5 mm (median 2.3, sd 1.7) (Table 6).

**Table 6:** Peri-implant bone loss after 20 years

Implant type	*n*	Min. bone loss	Max. bone loss	Median	Mean	sd
Tissue-level conical	14	− 0.5	4.5	1.7	1.7	1.4
Bone-level butt-joint	41	0	7.2	1.9	2.3	1.7
Bone-level conical	38	0.4	7.4	2.7	3.0	1.6
Total	93	− 0.5	7.4	2.3	2.5	1.7

Presentation of minimal/maximal bone-level values at the end of the study (20 years) according to implant type

*sd*  standard deviation

### Bone loss (between 10 and 20 years)

Between the 10th and 20th years of intraoral function and under SIT conditions, we found a mean bone loss of 0.8 mm (median 0.7, sd 1.1). Not all of the investigated implants showed bone loss. In 10 implants (11%), a mean bone gain of 0.9 mm (median − 0.8, sd 0.3) was found. No PBL changes were found in 30 implants (32%) if the level changes in an interval of [− 0.5, 0.5] were regarded as no change, whereas the remaining 53 implants (57%) revealed a mean increase of bone loss of 1.6 mm (median 1.4, sd 0.8) (Table 7).

**Table 7:** Peri-implant bone loss between 10 and 20 years

Implant type	*n*	Min. bone loss	Max. bone loss	Median	Mean	sd
Tissue-level conical	14	− 1.5	3.2	0.2	0.5	1.4
Bone-level butt-joint	41	− 1.4	2.6	0.4	0.6	0.8
Bone-level conical	38	− 1.1	4.4	1.0	1.1	1.2
Total	93	− 1.5	4.4	0.7	0.8	1.1

Presentation of minimal/maximal bone loss values during the study (10 to 20 years) according to implant type

*sd*  standard deviation

### Different implant–abutment designs

For the three groups, we found mean bone loss values (between 10 and 20 years) of 1.1 mm (BLC), 0.6 mm (BLB) and 0.5 mm (TLC). Statistically, no significant differences were seen among the implant systems during the observational period.

### Different implant surfaces

Two subgroups were built depending on the type of implant surface and were statistically tested. While BLBs had machined surfaces, BLCs and TLCs showed roughened surfaces. No significant differences (*p* = 0.63) could be found for the 10-year values or the 20-year values (*p* = 0.096). During the observational period, we found mean bone loss values of 0.6 mm for machined implants and 1.0 mm for roughened implants. This difference did not reach statistical significance (*p* = 0.09).

### Preexisting increased bone loss

A subgroup was built with a mean change in PBL > 2 mm at study baseline (10 years). It comprised 47 implants with a mean change in PBL of 2.4 mm. During the observational period of this study (10 to 20 years), these implants showed an additional mean change in PBL of 1.35 mm, leading to a total change in PBL value of 3.74 mm after 20 years. The remaining group of 46 implants had a mean change in PBL of 0.95 mm after 10 years, an additional 0.28 mm during this study and 1.23 mm after 20 years. The statistical analysis revealed high significance (*p* < 0.0001), supporting the conclusion that implants with a change in PBL > 2 mm after 10 years have an accelerated risk for increased bone loss compared to implants with a change in PBL ≤ 2 mm after 10 years.

### Peri-implantitis

During the observational period, 23 implants were diagnosed with peri-implantitis. After 10 years, these patients showed a mean change in PBL of 2.4 mm (median 2.1, sd 1.9), and after 20 years, we found a mean change in PBL of 3.0 mm (median 2.5, sd 2.3). The remaining 70 ‘healthy’ implants had a 10 year mean change in PBL of 1.5 mm (median 1.1, sd 1.2) and a 20 year mean change in PBL of 2.3 mm (median 2.2, sd 1.4). This difference was significant after 10 years (*p* = 0.01) but not after 20 years (*p* = 0.11) or for PBL values during the observational period (*p* = 0.52).

## Discussion

### Main results

**Fig. 1:**
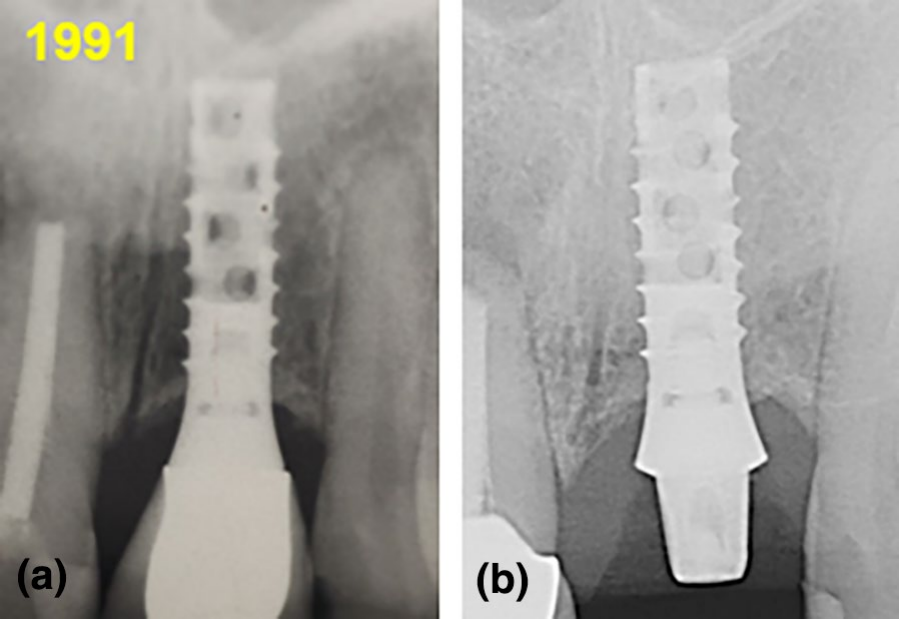
**a**, **b** Bone-level changes around a two-part tissue-level implant; from baseline 0 years, (**a**) to > 20 years of intraoral function, no bone loss was noted, but a small amount of bone gain might be identified

**Fig. 2:**
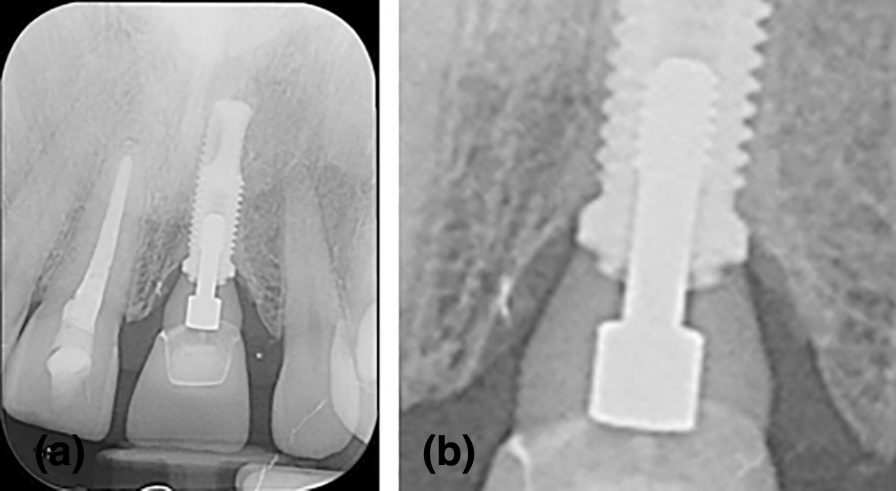
**a**, **b** Subcrestally placed bone-level butt-joint implant with external hex connection (**a**) in a 10-year control (**b**)

**Fig. 3:**
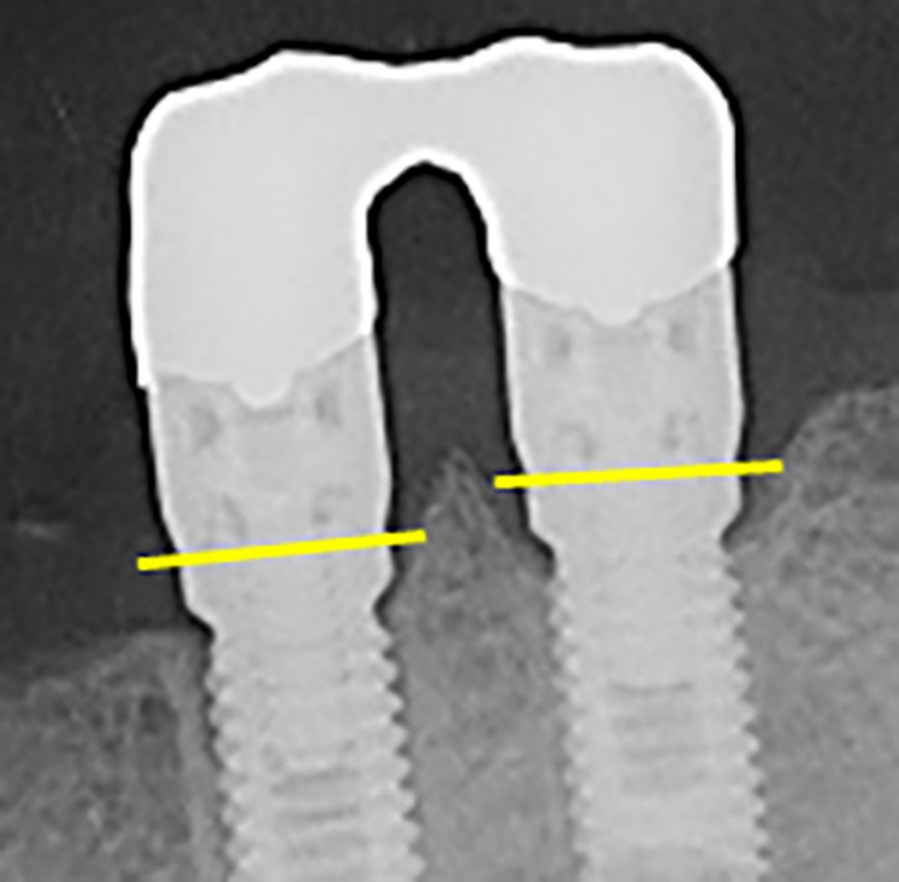
Bone-level implants with an external hex connection in a 10-year control displaying typical circular peri-implant bone loss (yellow lines represent the level of the implant–abutment connection)

**Fig. 4:**
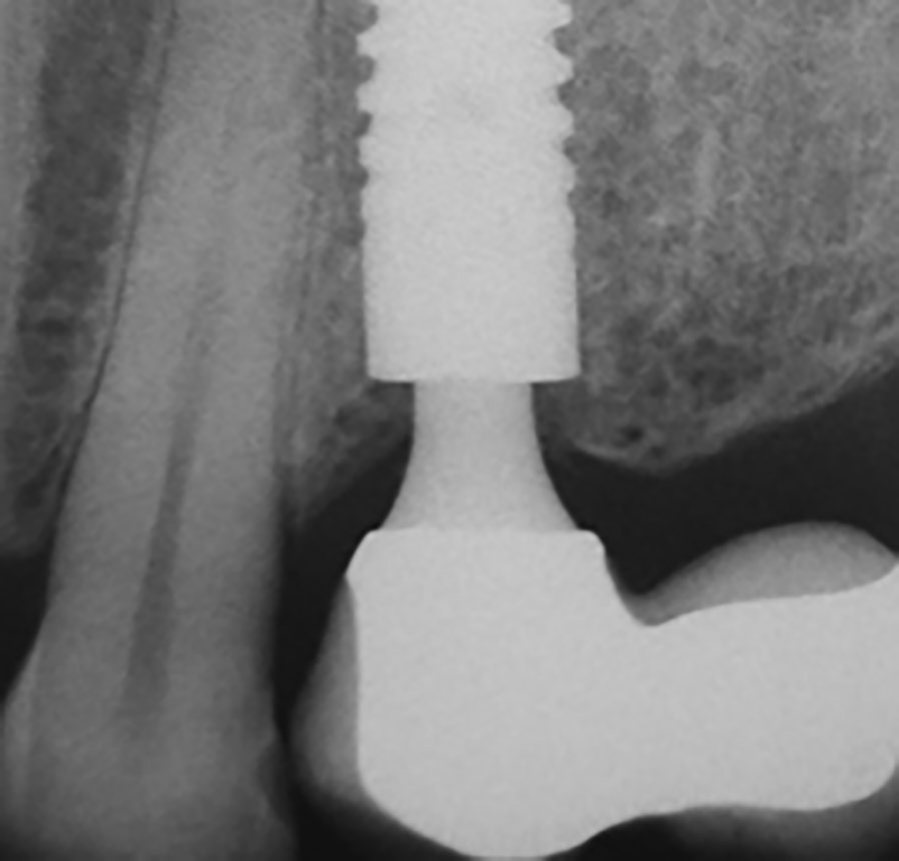
Subcrestally placed bone-level conical implant with a morse-taper connection in a 10-year control: no peri-implant bone loss can be found

The present study comprised 36 patients with 93 dental implants under conditions of an SIT program in a private practice setting from the 10th to 20th year of intraoral service. We assessed the peri-implant bone-level changes during the 2nd decade of function and found a median change in PBL of 0.7 mm and a mean change in peri-implant bone level of 0.8 mm. In the first investigation, three subgroups were built according to the implant–abutment connection concepts (BLC, BLB, and TLC; Figs. 1, 2, 3, 4). In the statistical analysis, no significant differences in peri-implant bone level were found. In a second investigation, two subgroups were built according to the different implant surfaces (machined vs. roughened), which also showed no significant differences after 10 and 20 years.

### Interpretation

The criteria for acceptable values of peri-implant bone loss introduced by Albrektsson et al. were < 1.5 mm during the first year and < 0.2 mm during each following year based on the knowledge of Branemark implants [15]. Transferred to a 20-year observational period, this would result in an ‘acceptable’ change in peri-implant bone level of up to 5.3 mm in total, which seems to be quite extensive. Since then, a variety of studies on peri-implant bone-level changes have been published.

After implant placement and after installation of the implant-supported prostheses, peri-implant bone remodeling processes are inevitable. This remodeling starts from the moment when the implant is installed (one-part/tissue-level implant types) or from the moment of implant uncovering surgery (two-stage BL implant types) when the implant/abutment connection is exposed to the intraoral microbial biofilm [21]. From this moment on, the peri-implant soft tissues (epithelium and subepithelial connective tissue) are in contact with the implant and/or abutment surface, and the biologic width is established [22–24].

To date, two different types of dental implants have been used. Two-piece implants are placed at BL height. Usually, they have to be uncovered in a second surgery, and then, the abutment is installed. In contrast, one-piece implants are placed at or above the soft-tissue level and, therefore, contain a part that contacts the soft tissue. They do not require second-stage surgery. In a comparison of the two types of implants, no significant differences in the vertical dimension of the biologic width were detected [24]. Another study compared three different implant systems: hex connection/BL, morse-taper connection/BL and conical connection/tissue level. No significant differences were found; the junctional epithelium had a height of 1.5–2 mm, and the connective tissue had a height of 1–2 mm [25].

However, the installation of the implant abutment at the BL implicates a microgap or microleakage between the two components that is accessible for the sulcus fluid and, therefore, for the intraoral microbia as well [26, 27]. Consequently, a biological reaction of the human host leads to the establishment of an inflammatory cell infiltrate [28]. In a review, Linkevicius and Apse found that there is ‘enough evidence … to state that the function of the biologic width around implants is to protect underlying bone’ [29]. Therefore, it seems likely that in cases of microgap-induced inflammation, the biologic width is established apically to the ‘infection’, which has to be realized via peri-implant bone resorption (1.5–2 mm circular) during the first period after loading. Implant types without microleakage (one-part implants, implant types with abutment connections at the soft tissue level and not at the BL) do not show this initial circular peri-implant bone loss (Figs. 1, 2, 3, 4). A recent review indicated that microleakage seems to be ‘very reduced in morse taper implants in comparison to other implant connections’ [30].

Ravald et al. compared the change in PBL between turned implants and roughened implants after 12–15 years in a retrospective study [31]. At 12 years after bridge installation, the authors found an annual change in the PBL value of 0.04 mm for the turned implants and that of 0.07 mm for the roughened implants. In the present study, the patients displayed an annual change in PBL of 0.08 mm during years 10 to 20, which seems to be within the same range.

In a prospective study design, Vervaeke et al. assessed 39 patients with 243 implants after 9 years of function, and they found a mean change in PBL of 1.7 mm using implant installation as baseline [32]. This represents exactly the same 10-year baseline PBL value of the present study.

In a 20-year life table analysis of a longitudinal study of > 12,500 implants with a roughened surface and a morse-taper connection, Krebs et al. found relatively low rates of bone loss [3]. After 204 months, 135 implants were assessed. Overall PBL values were not indicated, but 115 implants (85.2%) displayed a vertical bone loss of ≤ 1 mm, and 11 (8.1%) showed a change in PBL ≥ 2 mm. Our findings referring to the same implant system were different. We found a mean bone loss of 1.87 mm after 10 years and a mean change in PBL of 3.0 mm after 20 years.

Radiographic bone gain: In long-term studies, not all implants followed display peri-implant bone loss. In our investigation, 10.8% of the implants showed radiographic bone gain (RBG). This is in accordance with the findings of Roos-Jansaker et al., who analyzed 999 implants after 9 to 14 years [33]. Discounting the change in PBL during the first year, they found RBG in 10.7% of the implants.

### Limitations

Unfortunately, comparable studies with similar designs and observational periods were scarce. Due to the long observational period and to the retrospective character of the present study, it was not possible to create a control group. Moreover, no information concerning dropouts were available. Finally, only 36 patients could be comprised. Therefore, the validity of the presented results is obviously limited. Consequently, these findings will have to be verified by other researchers, greater patient samples, in different settings and including control groups. The fact that only one experienced periodontal surgeon performed all treatment steps may represent another limitation. It should be stated that the present results were assessed under professional SIT conditions and, therefore, must not be transferred readily to other patient conditions.

## Conclusions

In a private practice setting and under conditions of a systematic SIT program, clinicians may expect mean values of 0.08 mm per year of peri-implant bone loss in the 2nd decade of function. SIT programs might be helpful for long-term peri-implant bone-level preservation.

No significant differences were found between the three different implant–abutment connection concepts or different surfaces. Patients should be monitored intensively for a possible diagnosis of peri-implantitis leading to a respective treatment.

## Data Availability

The data sets used and/or analyzed during the current study are available from the corresponding author on reasonable request.

## Abbreviations

BLB: Bone-level butt-joint
BLC: Bone-level conical
BOP: Bleeding on probing
CHX: Chlorhexidine gluconate
FDI: Federation Dentaire Internationale
FGG: Free gingival graft
KM: Keratinized mucosa
max.: Maximal
min.: Minimal
PBL: Peri-implant bone level
PECTG: Partially epithelialized free connective tissue graft
PD: Probing depth
RBG: Radiographic bone gain
sd: Standard deviation
SIT: Supportive implant therapy
TLC: Tissue-level conical
